# Quantitative image analysis in COVID-19 acute respiratory distress syndrome: a cohort observational study.

**DOI:** 10.12688/f1000research.75311.2

**Published:** 2022-05-24

**Authors:** Tamas Dolinay, Dale Jun, Abigail Maller, Augustine Chung, Brandon Grimes, Lillian Hsu, David Nelson, Bianca Villagas, Grace Hyun J Kim, Jonathan Goldin

**Affiliations:** 1 Department of Medicine, University of California, Los Angeles, Los Angeles, CA, 90095, USA; 2 Department of Medicine, Barlow Respiratory Hospital, Los Angeles, CA, 90026, USA; 3 Department of Radiology, University of California, Los Angeles, Los Angeles, CA, 90095, USA

**Keywords:** acute respiratory distress syndrome, COVID-19, quantitative high-resolution lung CT scanning

## Abstract

*Background*

Acute respiratory distress syndrome (ARDS) is a severe form of acute lung injury commonly associated with pneumonia, including coronavirus disease-19 (COVID-19). The resultant effect can be persistent lung damage, but its extent is not known. We used quantitative high resolution computed tomography (QHR-CT) lung scans to radiographically characterize the lung damage in COVID-19 ARDS (CARDS) survivors.

*Methods*

Patients with CARDS (N=20) underwent QHR-CT lung scans 60 to 90 days after initial diagnosis, while hospitalized at a long-term acute care hospital (LTACH). QHR-CT assessed for mixed disease (QMD), ground glass opacities (QGGO), consolidation (QCON) and normal lung tissue (QNL). QMD was correlated with respiratory support on admission, tracheostomy decannulation and supplementary oxygen need on discharge.

*Results*

Sixteen patients arrived with tracheostomy requiring invasive mechanical ventilation. Four patients arrived on nasal oxygen support. Of the patients included in this study 10 had the tracheostomy cannula removed, four remained on invasive ventilation, and two died. QHR-CT showed 45% QMD, 28.1% QGGO, 3.0% QCON and QNL=23.9%. Patients with mandatory mechanical ventilation had the highest proportion of QMD when compared to no mechanical ventilation. There was no correlation between QMD and tracheostomy decannulation or need for supplementary oxygen at discharge.

*Conclusions*

Our data shows severe ongoing lung injury in patients with CARDS, beyond what is usually expected in ARDS. In this severely ill population, the extent of mixed disease correlates with mechanical ventilation, signaling formation of interstitial lung disease. QHR-CT analysis can be useful in the post-acute setting to evaluate for interstitial changes in ARDS.

## Introduction

Acute respiratory distress syndrome (ARDS) is a severe form of lung injury requiring intensive care unit (ICU) hospitalization. The etiology of ARDS is broad, but approximately 40% of cases are complications of respiratory infections ^1^. Despite modern intensive care the mortality of ARDS remains above 30% ^2, 3^ and international statistics show that ARDS is responsible for approximately 10% of all ICU admissions ^3^. During the 2020–2021 coronavirus disease (COVID)-19 pandemic, ARDS emerged as a feared complication of COVID-19 pneumonia driving ICU hospitalizations and mortality ^4, 5^. Clinical data also suggest that recovery from COVID-19-associated ARDS (CARDS) is prolonged requiring ongoing respiratory support beyond what is traditionally seen in ICU care ^6, 7^.

High resolution-computer tomography (HR-CT) scans with quantitative analysis (QHR-CT) have been widely used to study the details of the lung parenchyma ^8, 9^ and have been beneficial in tracking the progression of interstitial lung disease ^10^. In ARDS, the use of CT analysis was initially hindered by concerns over transportation of the critically ill. However with portable and faster CT scanners available in routine clinical care, HR-CTs have proved its usefulness in determining alveolar damage and edema formation ^11, 12^. Less is known regarding its utility in following the clinical course of the disease as lung infiltrates seen in the acute phase of ARDS usually resolve ^13^. In spite of this, in a minority of patients, pulmonary fibrosis may develop which has been associated with poor outcome ^14^. Recently McGroder *et al*. and in a separate analysis Gonzalez *et al*. reported persistent lung infiltrates and fibrosis-like changes 3–4 months after severe COVID pneumonia ^6, 7^. This may suggest that persistent lung changes in ARDS are more common than initially reported.

In our study, we examined the extent of persistent lung changes in CARDS survivors requiring continued hospitalization. Our findings suggest ongoing lung damage, which may give rise to pulmonary fibrosis.

## Methods

### Patient enrollment

Our study was approved by the Western Institutional Review Board (IRB) protocol ID #20210635. In this single center observational cohort study, patients admitted to Barlow Respiratory Hospital (BRH) with a diagnosis of CARDS were considered for enrollment. To avoid selection bias, we approached consecutively admitted patients to participate in the study. BRH is a non-profit long-term acute care hospital (LTACH) serving the greater Los Angeles area. Patients are transferred to BRH for ongoing respiratory care from short-term acute care hospitals (STACH). CARDS diagnosis was made during STACH hospitalization based on the following criteria: 1. at least one positive COVID-19 PCR test on admission to STACH, 2. new bilateral lung infiltrates in the past seven days on chest imaging not attributed to pulmonary edema alone, 3. requirement for invasive or non-invasive mechanical ventilation with at least 5 cm H _2_O positive pressure, 4. ratio of partial arterial oxygen pressure (PaO _2_) and fraction of inspired oxygen (FiO _2_) <300.

To participate in the study, informed written consent was obtained directly from the patient by the investigators. In case, the patient was not directly consentable, per the IRB recommendations, consent was obtained from the patient’s power of attorney. We enrolled 25 patients in our study between February and July 2021. Patients were consented to undergo lung QHR-CT during BRH admission. From the 25 enrolled patients, 20 completed the CT scans and their demographic and clinical data was collected by chart review. Data was entered in a password protected database. We did not record data of the patients, who did not complete the CT scans to avoid bias from an incomplete dataset.

The original QHR-CT scans and database contain sensitive patient information and is not publicly available for review per IRB guidelines. We created a deidentified database to share with the readers, which contains all pertinent patient demographics, clinical data, QHR-CT scan scores and volume measurements. This database is uploaded in a public database ^15^.

### Demographics and clinical data

Patients’ age, gender, race, ethnicity, STACH admission date, LTACH admission date, CT scan date, premorbid medical condition, tracheostomy status on admission, presence or absence of mechanical ventilation and mechanical ventilation mode were collected at the time of LTACH admission. The following premorbid conditions were considered: bacterial pneumonia, pneumothorax, acute or chronic kidney disease, hypertension, diabetes mellitus, deep vein thrombosis (DVT), pulmonary embolism (PE), heart failure, coronary artery disease (CAD), cerebrovascular accident (CVA), obesity with body mass index greater than 30 and pulmonary fibrosis. During LTACH stay data was collected for inpatient death, need for continued mechanical ventilation, on tracheostomy decannulation status and fraction of inspired oxygen (FiO _2_) on discharge.

### Image analysis

HR-CT lung scans were performed using a General Electric BrightSpeed 16 slice CT scanner (Model # 5128609-2, General Electric Health Corporation, Chicago, IL, USA) with 2mm cuts at BRH. QHR-CT analysis was performed by University of California Los Angeles (UCLA) Radiology department. Quantitative scores were measures for four distinct radiological patterns: 1. ground glass opacity, (QGGO), 2. mixed diseases (QMD), 3. consolidation (QCON) and 4. normal lung (QNL). The sum of three abnormal lung tissue scores was named the quantitative total lung diseases (QTLD). We applied the domain adaptation for calculating quantitative COVID-19 scores from HR-CT images. Quantitative lung scores were expressed as percent of predicted total lung capacity (% of TLC) calculated from HR-CT. To calculate the % of TLC, we used the formula (TLC volume from HR-CT)/((7.99*height)-7.08)*100 for men and (TLC volume from HR-CT/((6.6*height)-5.79)*100 for women. The source data and technique were adapted from the previously developed algorithm for diffuse lung disease ^16, 17^ and the target data was HR-CT images containing consolidation ^18^. The final model was reviewed and visually confirmed using an independent COVID-19 cohort at UCLA. Ground glass opacities usually represent acute inflammatory processes, mixed disease is commonly a radiological presentation of interstitial lung disease (ILD) and consolidation is frequently associated with pulmonary infection ^19^.

### Mechanical ventilation

Based on the need for mechanical ventilation at LTACH admission, three groups of patients were created: 1. mandatory mode mechanical ventilation via tracheostomy (MV), 2. spontaneous mode mechanical ventilation via tracheostomy (SV) and no need for mechanical ventilation (NV). MV included volume control ventilation, pressure control ventilation, and synchronized intermittent mandatory ventilation. SV included pressure support ventilation.

### Statistics

Patient characteristics and mechanical ventilation data were expressed as a percentage of total. Age and FiO _2_ were expressed as mean± standard deviation (SD). The lung disease score was expressed as % of TLC± interquartile range (IQR). The extent of mixed disease (QMD) was expressed as QMD/TLC± IQR and QMD/QTLD± IQR. QMD was correlated with the presence of mechanical ventilation on admission, tracheostomy decannulation. Statistical analysis was performed using non-parametric Kruskal-Wallis equality-of-population rank test with Bonferroni correction for the multiple comparisons. p < 0.05 was considered statistically significant. Correlation coefficient was calculated between the discharge FiO _2_, QMD and QMD/QTLD and data is shown with 95% confidence interval (95%CI). Stata 14.1 software (College Station, Texas 77845 USA, RRID:SCR_012763) was used for statistical analysis.

## Results

### Patient characteristics

We analyzed the CT images of 8 female and 12 male patients. The mean age was 61.2 years. There were 2 Asian, 1 American Indian/Native Alaskan, 3 African American, 6 Latino and 8 White patients in our cohort. Twelve patients were Non-Hispanic and 8 patients were Hispanic. At the time of LTACH admission 16 patients had tracheostomy and required invasive mechanical ventilation, 2 patients arrived on high flow oxygen support and 2 patients needed low flow oxygen. Of the 20 patients, 16 patients had secondary bacterial, 7 patients had pneumothorax secondary to mechanical ventilation, 9 patients had renal disease, 12 had hypertension, 12 had diabetes mellitus, 6 had DVT, 2 had PE, 3 patients suffered from heart disease, 3 had recent CVA, 9 were obese and none of the patients had a prior diagnosis of pulmonary fibrosis. Demographics and premorbid conditions are shown in Table 1.

**Table 1.** Patient demographics and premorbid conditions on LTACH arrival.

Characteristic	N = 20
Gender, Female N (%)	8 (40.0)
Age, Mean (SD)	62.1 (10.3)
Race, N (%)	
American Indian/Native Alaskan	1 (5.0)
Asian	2 (10.0)
Black/African American	3 (15.0)
Hispanic/Latino	6 (30.0)
White	8 (40.0)
Ethnicity, N (%)	
Hispanic	8 (40.0)
Non-Hispanic	12 (60.0)
Premorbid condition	
Bacterial pneumonia N (%)	16 (80)
Pneumothorax N (%)	7 (35)
Kidney disease N (%)	9 (45)
Hypertension N (%)	12 (60)
Diabetes mellitus N (%)	12 (60)
DVT N (%)	6 (30)
PE N (%)	2 (10)
Heart failure N (%)	3 (15)
CAD N (%)	2 (10)
CVA N (%)	3 (15)
Obesity N (%)	9 (45)
Pulmonary fibrosis N (%)	0 (0)

### Respiratory support

Specifics of airway management, mechanical ventilation and liberation from mechanical ventilation are listed in Table 2 and can also be found in the online database. Sixteen patients required invasive mechanical ventilation via tracheostomy on LTACH admission. Of those 13 arrived with MV and 3 with SV. Four patients needed supplementary oxygen via nasal cannula, 2 with high flow and 2 with low flow (less than 10 liters per minute) systems. Twelve patients were liberated from mechanical ventilation and 10 patients had the tracheostomy cannula removed (decannulated) before LTACH discharge. Two patients needed continued MV on discharge. Two patients died during the LTACH stay. No patients required resumption of mechanical ventilation. Five patients were discharged on room air. The average supplementary FiO _2_ on discharge was 27.6% (SD = 6.8).

**Table 2.** Respiratory support in CARDS patients.

	N = 20
**Respiratory support on admission**	
VC N (%)	10 (50)
PCV N (%)	3 (15)
PS N (%)	3 (15)
High flow nasal cannula N (%)	2 (10)
Low flow nasal cannula N (%)	2 (10)
Tracheostomy N (%)	16 (80)
**Respiratory support on discharge**	
Decannulated N (%)	10 (50)
VC N (%)	2 (10)
Low flow nasal cannula N (%)	13 (65)
No respiratory support N (%)	5 (25)
FiO _2_ % Mean (SD)	27.2 (6.8)
Inpatient mortality N (%)	2 (10)

***Quantitative lung injury scores correlate with disease severity on admission***

**Figure 1.**
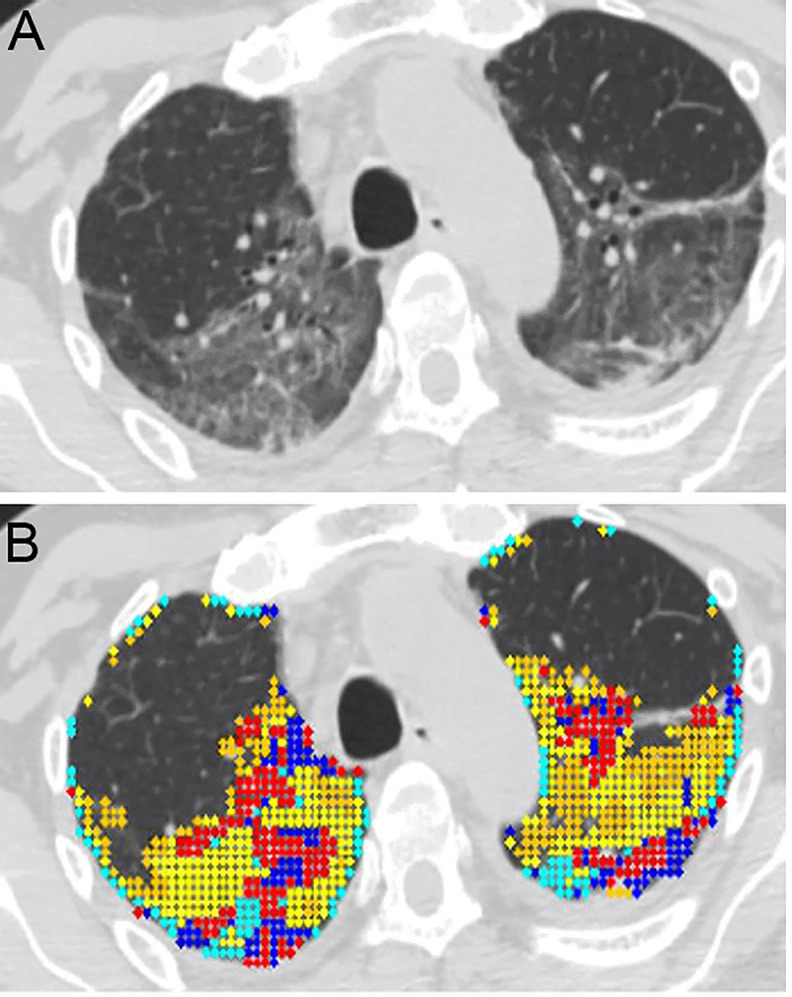
Quantitative high-resolution CT analysis of COVID-19 acute respiratory distress syndrome (ARDS). **A**. Horizontal high resolution (HR)-CT lung cut of a selected COVID-19 ARDS (CARDS) patient. The patient is a 61-year-old male with mandatory mechanical ventilation on arrival to the long-term-acute care facility. **B**. Color coded quantitative HR-CT (QHR-CT) analysis in CARDS. Quantitative scores were 19.1% mixed disease (QMD), 25.1% of ground glass opacities (QGGO) and 3.4% consolidation (QCON). The ratio of QMD to quantitative total lung diseases (QTLD) was 40.1%. Red and blue = mixed disease (QMD), yellow and cyan = ground glass opacities (QGGO), peach = consolidation (QCON).

QHR-CT was performed between two and three months post diagnosis of CARDS. The average predicted TLC was low, 2175.3 ml (SD=574.1), mean % of TLC=38.48 (SD=13.9). There was significant persistent lung damage with QTLD = 76.1% (IQR=15.6) of TLC. The majority of lung pathology was QMD with 45.0% (IQR=24.7) of TLC. QGGO was 28.1% (IQR=9.6), QCON was 3.0% (IQR=4.5) with little normal lung tissue remaining QNL = 23.9% (IQR=15.6). The distribution of lung disease was equal in both lungs (data not shown). Table 3 shows the extent of lung disease in relation to TLC. Figure 1A shows an example of the distribution of lung disease on CT scan and Figure 1B depicts the patterns of lung abnormalities in color coded fashion.

**Table 3.** Quantitative HR-CT scores by pattern in CARDS.

QHRCT score	% of TLC (IQR)
QGGO	28.1 (9.6)
QMD	45.0 (24.7)
QCON	3.0 (4.5)
QTLD	76.1 (15.6)
QNL	23.9 (15.6)

The extent of QMD was correlated with admission use of respiratory support ( Table 4). Patients with MV and SV had more QMD% than their NV counterparts. When QMD/QTLD ratio was calculated, MV patients had significantly higher ratio than NV patients (p < 0.0127). There was no correlation between the extent of QMD and tracheostomy decannulation or need for supplementary oxygen on discharge ( Table 4).

**Table 4.** The relationship between quantitative mixed disease score and respiratory support on LTACH admission and discharge.

	QMD scores (% of TLC), mean (IQR)	QMD/QTLD, mean (IQR)
**Respiratory support on admission**		
MV (N = 13)	50.4 (17.9)	0.6 (0.1)
SV (N = 3)	41.4 (40.4)	0.5 (0.3)
NV (N = 4)	30.3 (10.5)	0.4 (0.1) *
**Tracheostomy decannulated before discharge**		
Yes	48.4 (17.9)	0.6 (0.1)
No	49.2 (24.4)	0.6 (0.2)
Use of supplementary FiO _2_ on discharge	QMD scores (% of TLC), Correlation Coefficient (95% CI)	QMD/QTLD, Correlation Coefficient (95% CI)
Yes	0.264 (−0.231 – 0.651)	0.327 (−0.165 – 0.689)

* Represents significant difference between MV and NV, p = 0.0127, Kruskal-Wallis test; IQR=interquartile range

## Discussion

Modern ICU care significantly improved the immediate survival of ARDS, but little is known about the long-term respiratory complications of the disease ^3, 20^. Herridge *et al*. reported that 20% of ARDS survivors have abnormal chest imaging at one year ^21^ and more recently Burnham *et al*. showed that 25% ARDS survivors may have ILD at six months ^22^. The etiology of persistent interstitial lung changes post ARDS is not well understood, but it has been associated with poor quality of life ^14, 22^. During the COVID-19 pandemic, ARDS cases soared ^5^ and preliminary studies suggest that 30-40% of critically ill COVID-19 patients have persistent lung changes ^6, 7^. It has been long speculated that a major factor for the development in ILD in ARDS is invasive positive pressure mechanical ventilation (commonly abbreviated mechanical ventilation) ^23^. Although lifesaving, mechanical ventilation has been associated with increased rates of pulmonary fibrosis post ARDS ^14^. Recently McGroder *et al*. showed fibrosis-like radiographic changes in 72% patients receiving mechanical ventilation compared to 20% of non-ventilated COVID-19 patients ^6^. The etiology of fibrosis is unclear but our imaging analysis in agreement with other studies that describe a non-specific post-inflammatory origin ^7, 14^. All together this data suggests that survivors of CARDS maybe prone to ILD and pulmonary fibrosis thus, requiring long-term monitoring ^24^.

In our study we performed QHR-CT lung analysis of CARDS survivors, who require ongoing respiratory care two to three months after the initial diagnosis. LTACH patients represent a unique population of the chronically critically ill with significant morbidity and mortality. We have previously reported that 80% of COVID-19 patients requiring LTACH admission have tracheostomy and 51% are receiving mechanical ventilation ^25^. In this severely ill population, it is difficult to apply lung function testing and quality of life questionnaires to assess respiratory status. QHR-CT has been useful in following patients with ILD and can detect disease progression ^10^. HR-CT can be relatively easily performed in non-cooperative patients and quantitative analysis provides insight to the ongoing lung disease. We found that in our population of CARDS patients, there was significant lung disease involving, on average, 76% of the lungs. The most significant form of lung changes (45%) were consistent with mixed disease, which is a combination of reticulation and traction bronchiectasis, suggesting ILD. ILD changes usually result in permanent scaring and can lead to pulmonary fibrosis. In comparison, ground glass opacities and consolidation seen with acute inflammation and infections usually resolve. These findings suggest that ILD is more common in CARDS than other forms of ARDS. We also observed that patients who did not require mechanical ventilation on admission, had less ILD, which is consistent with findings of McGroder *et al*. ^6^ However, we did not find association between the extent of mixed disease and tracheostomy decannulation or need for supplementary oxygen at discharge. This data signals that the cession of mechanical ventilation or lower oxygen supplementation will not reverse the damage that has already occurred.

Our study has several strengths: 1. it studies a chronically critically ill population, in which the outcomes of ARDS are not known, 2. it shows that QHR-CT can be easily used to study lung disease in a population where traditional respiratory tests are difficult to perform, 3. it adds to our understanding of ILD development post ARDS. However, our study also has limitations: 1. it studies a small group of CARDS patients in a single hospital in Los Angeles, California, which may limit generalizability; 2. CARDS is a complex disease and our limited dataset allowed only the analysis of a select number of respiratory parameters, which may not have taken into account other possible confounders in our analysis; 3. QHR-CT technology is not specific to ARDS and some of the unique pathologic and radiographic changes of this disease may have been missed; 4. we did not perform serial imaging and we do not know, if the observed mixed disease, will progress with time; 5. lastly, we used HR-CT technology which results in radiation exposure.

## Conclusions

In conclusion, our study suggests that lung disease is highly prevalent in CARDS two to three months after the initial infection. ILD is the most prominent findings on imaging, which may result in progression to fibrotic disease. We recommend following CARDS patients with HR-CT beyond the acute care setting to evaluate for the development of ILD.

## Data availability

### Underlying data

OSF: CT in COVID https://doi.org/10.17605/OSF.IO/S2FXN ^15^.

The project contains the following underlying data:

- CT database-deidentified_final_with_CT_scores_11-18-21.xlsx

Data are available under the terms of the Creative Commons Zero “No rights reserved” data waiver (CC0 1.0 Public domain dedication).

## Consent statement

Informed written consent was obtained directly from the patient by the investigators. In case, the patient was not directly contactable, per the IRB recommendations, consent was obtained from the patient’s power of attorney.
