# Supplementary material for: Quantitative image analysis in COVID-19 acute respiratory distress syndrome: a cohort observational study
Source: F1000Res. 2023 Mar 31;10:1266. Originally published 2021 Dec 9. [Version 3] doi: 10.12688/f1000research.75311.3 (PMC10182379; doi:10.12688/f1000research.75311.3)
Supplement: Peer review history

# Peer review: Reviewer response for version 3

**DOI:** 10.5256/f1000research.146057.r168408

**Published:** 2023-05-12

**Authors:** Prashant Nasa

**Affiliations:** 1 Head Critical Care Medicine, NMC Specialty Hospital, Dubai, United Arab Emirates

I agree with the reply comments by the authors and do not have any new comments.

Is the work clearly and accurately presented and does it cite the current literature?

Yes

If applicable, is the statistical analysis and its interpretation appropriate?

Yes

Are all the source data underlying the results available to ensure full reproducibility?

Yes

Is the study design appropriate and is the work technically sound?

Yes

Are the conclusions drawn adequately supported by the results?

Yes

Are sufficient details of methods and analysis provided to allow replication by others?

Yes

Reviewer Expertise:

NA

I confirm that I have read this submission and believe that I have an appropriate level of expertise to confirm that it is of an acceptable scientific standard.

# Peer review: Reviewer response for version 2

**DOI:** 10.5256/f1000research.134257.r164894

**Published:** 2023-03-20

**Authors:** Prashant Nasa

**Affiliations:** 1 Head Critical Care Medicine, NMC Specialty Hospital, Dubai, United Arab Emirates

I was privileged to review a manuscript on quantitative image analysis in COVID-19 related ARDS cohort observational study.

 The manuscript is very well written, and the research topic is engaging. The major limitation of this study is its small size, which should be acknowledged in the conclusion and abstract. The results from this study must be validated in a prospective larger cohort.

 There are minor concerns.

1. In the criteria for diagnosis of CARDS, the second criteria mention the use of at least 5 cm of H2O positive pressures. Instead, it should be corrected to at least 5 cm of H2O positive end-expiratory pressure. 
2. 4 out of 20 patients were on low or high-flow oxygen. These patients cannot be classified into ARDS criteria. Were these patients needed ventilatory support in the short-term acute care facility?
3. The CT findings of mixed disease in the discussion suggest progression to interstitial lung disease. Isolated findings alone cannot be used for the diagnosis of interstitial lung disease in these patients. Many of these radiographic findings entirely resolved over a duration ^1^. Close monitoring is advocated with imaging is advocated to evaluate the progression or persistence of these findings. 

Is the work clearly and accurately presented and does it cite the current literature?

Yes

If applicable, is the statistical analysis and its interpretation appropriate?

Yes

Are all the source data underlying the results available to ensure full reproducibility?

Yes

Is the study design appropriate and is the work technically sound?

Yes

Are the conclusions drawn adequately supported by the results?

Yes

Are sufficient details of methods and analysis provided to allow replication by others?

Yes

Reviewer Expertise:

COVID-19 related ARDS

I confirm that I have read this submission and believe that I have an appropriate level of expertise to confirm that it is of an acceptable scientific standard, however I have significant reservations, as outlined above.

## References

[1] : One-year follow-up CT findings in COVID-19 patients: A systematic review and meta-analysis. *Respirology* .2022;27(8) : 10.1111/resp.14311 605-616 10.1111/resp.14311 35694728PMC9350074

# Response

**Published:** 2023-03-23

**Authors:** Tamas Dolinay

**Affiliations:** UCLA Department of Medicine, USA

We would like to thank the reviewer for the useful comments. Please see below our point-by-point response:

1. In the revised manuscript, we corrected the definition of ARDS and added 5 cmH2O positive end-expiratory pressure.
2. We amended our Patients enrollment description to include that all patients had met ARDS diagnosis criteria prior to study enrollment, which included positive pressure ventilation.  
3. Thank you for this important question. We agree with the reviewer that the frequency pulmonary fibrosis and interstitial lung disease (ILD) post-ARDS are not known and further research is needed to study these questions. The lack of data is likely related to the facts that 1) there are multiple disease mechanisms resulting in ARDS and 2) there is no definition for post-ARDS chronic lung disease. Chronic lung changes are commonly described in COVID-19-associated ARDS (CARDS), as pointed out in the reference provided, and a growing body of literature suggests that the resolution of interstitial lung changes maybe slower in CARDS than in other forms of ARDS.  We agree with Watanabe and colleagues that “fibrotic-like changes” that can be seen in CARDS are not equal to “COVID-related fibrosis”, but can be recognized as risk factor for development of fibrosis.  However the mixed disease, marked by reticulation and traction bronchiectasis on qualitative- high resolution CT scanning (QHR-CT) show the image findings of ILD. This does not equal to permanent lung fibrosis but can be a sign of the development of permanent lung scaring. We believe our findings are in concert with theirs and our publication adds to the literature that supports continued monitoring of patients post CARDS. We added the reference to the revised version of the manuscript.

# Peer review: Reviewer response for version 1

**DOI:** 10.5256/f1000research.79165.r135370

**Published:** 2022-05-03

**Authors:** Jeffrey Haspel

**Affiliations:** 1 Division of Pulmonary and Critical Care Medicine, Department of Internal Medicine, Washington University School of Medicine, St. Louis, MO, USA

Here, Dolinay and colleagues report the prevalence of radiographic abnormalities in a cohort of COVID-19 survivors admitted to their LTACH. They note a high prevalence of CT abnormalities 2-3 months post COVID, consistent with ILD, and based on this suggest the need for long term radiographic follow up.  

 Overall, this is an interesting report that, combined with reports sure to follow from other centers, will be useful in clarifying the natural history of severe COVID-19. 

 My comments are minor:

1. Since TLC is reported as "low" based on CT, it would be helpful to supply the %predicted TLC.
2. Since the use of Kruskal Wallis suggests the data presented are non-parametric it is probably more correct to report IQR rather than SD/SE in Table 3 and in the text (SD/SE usually refer to normally distributed data).

Is the work clearly and accurately presented and does it cite the current literature?

Yes

If applicable, is the statistical analysis and its interpretation appropriate?

Yes

Are all the source data underlying the results available to ensure full reproducibility?

Yes

Is the study design appropriate and is the work technically sound?

Yes

Are the conclusions drawn adequately supported by the results?

Yes

Are sufficient details of methods and analysis provided to allow replication by others?

Yes

Reviewer Expertise:

Pulmonary and Critical Care. Circadian biology. Inflammation

I confirm that I have read this submission and believe that I have an appropriate level of expertise to confirm that it is of an acceptable scientific standard.

# Response

**Published:** 2022-05-19

**Authors:** Tamas Dolinay

**Affiliations:** UCLA Department of Medicine, USA

We would like to thank the reviewer for his useful comments. Please see below our point-by-point response:

 1. In the revised manuscript, we included the percent of predicted total lung capacity (% of TLC) for our patients (N=20) with standard deviation (SD).

 2. We agree with the reviewer that presenting the data  with interquartile ranges (IQR) is more representative  when used with non-parametric testing. We now show IQR for both Table 3 and 4.
